# Simulating the overload of medical processes due to system failures during a cyberattack

**DOI:** 10.1186/s12911-025-02988-8

**Published:** 2025-04-23

**Authors:** Markus Willing, Simon Ebbers, Christian Dresen, Marc Czolbe, Christoph Saatjohann, Sebastian Schinzel

**Affiliations:** 1 Fraunhofer SIT, Fraunhofer Institute for Secure Information Technology SIT| ATHENE National Research Center for Applied Cybersecurity, Steinfurt, Germany; 2 University Medical Center East Westphalia-Lippe, Bielefeld, Germany; 3 University of Applied Sciences Muenster, Muenster, Germany

**Keywords:** Medical IT-security, Process simulation, Risk management, Medical processes

## Abstract

Today’s medical IT is more and more connected and network or IT system outages may impact the quality of patient treatment. IT outages from cyberattacks are particularly worrisome if attackers focus on those medical IT devices that are critical for medical processes. However, medical processes are primarily documented for the hospital employees and not for analyzing the criticality of any given human or medical IT resource. This paper presents a generic model for realistic, patient-focused simulation of medical processes. The model allows the simulation of cyber incidents, focusing on device outages or overload situations like mass casualty incidents. Furthermore, we present a proof-of-concept tool that implements the described model, enabling end-users to simulate their processes. The tool offers the ability to run with low detailed data for overview purposes and highly detailed data for fine-grained simulation results. We perform different scenario simulations for a sample hospital, including the acute phase of a ransomware attack, negative performance impacts due to the implementation of cybersecurity measures, and emergency plans for mass casualty incidents. In each scenario, the respective simulation resulted in a quantitative statement of how these scenarios affect overall process performance and show possible key factors supporting decision-making. We use real-world data from a German trauma room to optimize and evaluate the process simulation.

**Supplementary Information:**

The online version contains supplementary material available at 10.1186/s12911-025-02988-8.

## Background

Medical devices are critical for patient care and must be highly available and resistant to malfunctions. A wide range of different cooperating devices with specific applications combined with trained hospital personnel enables high-end medicine with the best possible outcome for patients. This medical equipment is not always available as it must be serviced regularly or may fail due to defects. From an organizational point of view, maintenance work can be planned and is part of hospital schedules and processes.

Defects, however, are failures caused by randomly distributed error conditions and are more critical because they occur randomly and are difficult to plan. Since the digitization process progresses in the healthcare sector, another factor can lead to outages of medical devices: Cyberattacks as shown in the Table 1. Those attacks are not predictable, and adaptive attackers can cause worst-case damage at any particular time. For example, the University Hospital of Düsseldorf, Germany, experienced a large-scale ransomware attack in September 2020. The following outages of various IT devices lead to a breakdown of emergency care and major parts of the hospital infrastructure.

**Table 1:** The German industry-specific security standard for proving the state of the art in healthcare lists these cyber threats for hospitals, among others [1]

	B3S: IT-Threats to hospitals
**1**	**General threats**
1a	E. g. Failure of basic infrastructure (Powersupply,‥)
**2**	**Vulnerabitilities**
2a	E. g. Use of unsuitable IT networks, linking of services
**3**	**Industry sector specific threats**
3a	E. g. Loss of confidentiality especially sensible patient data
**4**	**IT-specific threats**
4a	Not availibility of relevant data
4b	Not availibility of relevant IT-systems
4c	Not availibility of relevant logistic chains
4d	Manipulation of relevant data
4e	Hacking and Manipulation core systems
4f	Ransomware (or other malware)
4g	DDoS attacks
4h	Social Engineering
4i	Advanced Persistent Threat (APT)
4j	Identity Theft and missuse
4k	E-Mail Account theft

Even though the attackers had confused the Düsseldorf University Hospital with the University Düsseldorf and immediately stopped the attack a day later when they realized their attack had hit a hospital, it took four weeks to get the hospital to its normal capacity.

Nowadays, software patches, attack countermeasures, and further security measures are regularly implemented to improve cybersecurity and IT infrastructure resilience. Unfortunately, such changes can directly negatively influence medical processes and eventually on the patient’s well-being [2].

Processes in hospital environments are complex constructs with many dependencies that can lead to enormous loss of efficiency or failure of entire processes in case of a defect or a cyberattack [3]. For this reason, effective emergency plans are important, especially in the area of critical patient care. To create and validate such plans, precise risk management is necessary, including identifying critical pathways and components within emergency care. While there are various tools and tests to simulate processes and support such risk management in the industry, these tools cannot provide a simulation tailored to medical processes that maps to reality. Due to the recent increase of cyberattacks on hospitals and healthcare institutes, this research of simulating effects becomes more important [4, 5]. It is important to say, that not every major IT failure, that leads to a disruption of healthcare processes is caused by an attack. Especially in the early stages of an attack, the difference is sometimes not visible.

### Related work

Alemzadeh et al. showed that malfunctioning medical devices are one of the leading causes of serious injury and death in the US with 5.294 recalls and approximately 1.2 million adverse events reported to the FDA between 2006 and 2011. Computer malfunctions caused 23 % of these incidents, and 94 % presented medium to high risk of severe health consequences [6].

Spence et al. revealed that the number of successful ransomware attacks on healthcare facilities is growing. They claim that hospitals have to make substantial efforts to prevent such attacks and not risk financial and reputation loss [7]. However, Choi et al. showed that introducing security measures after a cybersecurity incident leads to a measurable negative effect on hospital care quality and patient outcomes [2].

In other industries, risk and process analysis are performed to control monetary losses and have already been extended to IT-related issues [8, 9]. It is done by abstracting the real-life process into a standard framework with defined steps of action [10, 11]. Process analysis in German hospitals became possible with the implementation of the German Diagnosis Related Groups System (G-DRG) system in 2003, where every medical procedure is categorized and accounted for with a specific flat rate [12, 13]. To analyze how vulnerable specific processes are to malfunction and accidents, workflow- and resource management became the focus of observational and post-mortem study approaches [14]. For example, results showed that physicians are prone to be interrupted and disturbed by multiple factors (e.g., nursing staff, phone calls) for 3.66 times per hour in a normal work shift [15].

BPMN, as a common model notation, provides a high usability and wide distribution throughout various fields of industries [16]. It provides a standard framework that allows not only the modeling of complex processes but also software systems [17]. Several studies [18, 19] also used this type of notation, with one showing the applicability of this notation in the department of emergency to discover bottlenecks [20]. Musman et al. [21] published a similar tool by introducing a decision-assisting and detailed assessment model that can be used for performing cyber risk analysis and crown jewels analysis. They also used BPMN as a standard notation framework for process modeling. Their objectives are partly transferable to our work in the context of clinical processes, as time and the achievement of various mission objectives also determine the outcome of a military mission. However, the survival of material and personnel is also a critical factor here.

The modeling and analysis of cyberattacks on organizations has several approaches in the literature. Various models have been proposed, which have different advantages and disadvantages in terms of applicability. Cohen compared in 1999 [22] the models of Howard [23] and Amaroso [24] but also defines the general purpose of such simulations. The limitation of all models is composed of the parameters: accuracy of the model, limits of the data accuracy on which the modeling is based, and the ability to explore the simulation space through the use of multiple runs of the simulation. Another younger, well-known source in the field of simulating and modeling cyberattacks is published by Kuhl et al. [25]. They propose a model of different attack scenarios with the outcome of several different intrusion detection alerts that can be used to evaluate cybersecurity systems.

### Contributions

We make the following contributions within this paper:

- We introduce a hybrid model of Discrete Event Simulation (DES) and Agend-Based Simulation (ABS) for hospital processes focusing on patient effects. It delivers results with a manageable configuration effort, which has the capacity of a variable detail level by further configuration and additional input data.
- We implement a simulation tool called SICKPATH. It allows to document relevant medical processes and their dependencies on resources such as staff, medical equipment, infrastructure, and hospital IT in a structured way and perform detailed analyses on how the total process runtime changes when critical resources are affected.
- We demonstrate the modeling and analysis capabilities of this simulation tool in a case of an acute phase of a ransomware cyberattack using sample simulations based on the process of an Emergency Trauma Room (ETR)

## Methods

The following section describes our approach, starting with the classification of the applied principle, followed by technical details of modeling and analyzing, the model definition, including the definition of a specific process, and concludes with our steps of optimization.

### Classification of the applied principles

Kuhl et al. used ARENA for simulating cyberattacks which is also the most popular tool with regards to simulation modeling in healthcare [26, 27]. ARENA implements the DES model which comes with divagates while simulation cyberattack scenarios in hospitals such as difficulties with non-linear dynamics and resource allocations [28]. These divagates can be addressed by combining DES models with the advantages of ABS model [29]. The hybrid approach to modeling offers an enhancement of realism by incorporating both individual decision-making and event-driven processes. Additionally, this hybrid model provides flexibility, as it can adapt to various scenarios, enabling the exploration of how individual behaviors influence system dynamics and vice versa. Finally, the hybrid model of DES and ABS improves decision support by providing valuable insights into how changes in individual behavior or system events can impact overall performance, thereby aiding in effective decision-making processes. Process mining is essential to make our hybrid model of DES and ABS of different scenarios as realistic as possible. Combining data mining approaches of historic data and insight from expert interviews, we were able to supplementary insights, validate simulation results, and contextualize the understanding during the process mining process. The real-world anonymized data set from the TraumaRegister DGU (TR-DGU) and the expert knowledge of employees of the Muenster University Hospital (UKM) made it possible to model areas and transport costs, available resources, and possible redundancies within the process. Furthermore, we identified patient and injury groups acting as agents during the simulation.

### Technical details

The technical implementation of this model can be divided into two parts, a web application that allows users to configure simulations and a *Python* [30] back end that implements the execution of simulations. The web application is constructed using the framework *Django* [31], which allows for straightforward access via a web browser. The framework is further enriched by *Javascript* [32] technologies and facilitates user-friendliness for non-technical users in interacting with the simulations. The execution of simulations, being computationally intensive, is queued as jobs and executed when computing resources are available. The web application and the part that executes the simulations are linked by two data stores. The first store is the relational database *MariaDB* [33] that contains the configurations and metadata such as user information. The second data store is the NoSQL search engine *Elasticsearch* [34], which holds the results of simulations. *Elasticsearch* is used for this instead of *MariaDB* as it scales better with the great amount of data that is generated by a simulation. The user initiates a simulation by queuing it into the job list of the first data store. The simulation is then executed by the back end that is listening for it. All the simulated information is stored in the second data store and can be accessed by the user via the web application in real time.

*Cybersecurity Scenarios* Cyberattacks against a hospital may range from single failures and reduced capacity of specific devices to large outages that affect multiple stations or even the entire hospital [35]. The effects correlate strongly with the degree of digitization and the availability of redundancies.

The model described in this work is designed to enhance cyber risk management using simulation of effects on process performance caused by cyberattacks. It enables risk management to learn which assets are critical within the processes, focusing on patient safety. This may be trivial for single and straightforward processes but hard for combinations of multiple complex processes with several dependencies: E.g. a process in an intensive care unit may depend on the availability of several different applications such as hospital information system, laboratory information system, networked medical devices as infusion pumps, X-Ray devices, ultrasonic devices and a number of staff with specific skills. An attack can negatively affect the performance of tasks inside a process. For example, a network failure can require the staff to transport data using USB sticks manually, slowing down the whole process. Further, it is essential to assess the maximum allowed duration of a recovery process from, for example, a ransomware attack to control the patient risk and rate mitigation measures. This, combined with the prioritization of critical assets within the processes, can optimize recovery and minimize patient risk. Besides attack and business continuity scenarios, the implementation of security measures may lead to an efficiency loss, often described as an increased process duration with more personal required [2]. Those emergency processes ensure basic process continuity. For example, if the hospital information system fails to be available, some hospitals provide an Offline fallback solution, which makes the latest status available locally even without a network connection to the databases. These effects should be considered before implementing security measures as they can affect the overall performance of critical processes up to direct influence on patient safety. We looked at several different real-world hospital processes and ended up choosing the ETR process because of its international standard and the availability of data, which is measured for quality reasons anyway. This makes the process comparable between different trauma departments.

*Virtual incident analysis - Creation of emergency plans* The reaction to incidents should be carried out as part of a Business Continuity Management (BCM) for specific scenarios. This can ensure a sufficient level of performance even under special conditions. The described model can be used in task forces to simulate various incidents individually and in combination, thus contributing significantly to the development and validation of emergency plans.

*Regional capacity planning* The emergency care capacities in Germany (1.35 emergency departments per 100,000 citizens) are the product of historically developed structures and federal planning [36, 37]. As a result, there are large differences in care capacity and quality. Urban and industrial areas have a higher density of large, well-equipped hospitals. In contrast, rural areas usually only provide basic care, and patients must be transferred to specialized centers in demanding cases [36]. The model described in this work and its technical implementation can simulate different patient volumes with locally different care areas. The model’s high potential depth is decisive for results with high relevance to reality. As an example, a simulation can provide information on how many trauma centers are necessary within a specific region to control a Mass Casualty Incident (MCI) of a defined size.

*Resource planning* Inside a hospital, shortages of resources such as specific devices, consumable materials, or specialized staff may occur, especially during emergencies or in high hospital-load situations like the COVID-19 pandemic. The behavior in the absence of individual and combined resources can be simulated to a high degree with the described model. This allows executives to assess the consequences of decisions in such situations regarding the effect on patient safety. For example, using this approach, it is possible to analyze how specific redundancies may improve the outcome of critical patients and the detection of capacity thresholds.

*Retrospective simulation of real-world events* In the case of a MCI, the medical and logistical staff of a trauma center should adhere to the plans provided for this purpose. However, real incidents can only be predicted up to a certain level of detail. In a wide variety of situations, it may be necessary for the personnel on-site to deviate from plans or make critical decisions to adapt to the specific situation. This can be, for example, because the situation had never occurred before and was therefore not taken into account when the plan was created. The model described in this work can be used for clinical supervision and to support classic crisis training scenarios by combining staff and technology with local conditions, such as building structures and patient logistics.

### Model definition

Hospital processes are often complex and rarely self-contained. They have a multitude of factors and dependencies which have to be considered for a realistic simulation. Following, we go into the factors and dependencies individually and show how these dependencies can be combined into a model.

*Process definition* A process is defined by one starting point, tasks in between, and one ending point. Between those two points, there are one or more branches of actions or sub-processes. Branches within a process can run conditionally or in parallel. Each action $A$ within a process ${P_r}$ consists of a list of required resource types $R$, a default usage duration per resource $D$, and an area ${A_r}$, e.g., a shock room or a radiology room.

Figure A.1 in Appendix A shows a simplified process from an ETR.

*Patients and Injury Groups* Every patient is assigned to an injury group $G$ and has a start time ${T_{start}}$. This value defines the point in time of their occurrence inside the process, e.g., the handover from the rescue service to the trauma room. Each group $G$ can be optionally configured with a range of values for Time till Damage (TTD) and Time to Reanimate (TTR) used during patient generation. The TTD describes the time until a patient takes irreversible damage if not treated. When this point is reached for a patient, the process is considered to have failed for that patient. Nevertheless, the patient continues to go through the process.

The TTR marks the time at which a patient requires cardio-pulmonary reanimation. This point in time is an end event in our simulation, and the patient is removed from the process because of the high variance of further treatment and duration, which cannot be modeled realistically. To further adapt the simulation to reality, a change in TTD and TTR depending on the group $G$ can be configured for each action $A$. For example, stopping heavy bleeding prolongs a patient’s TTD and TTR, whereas x-rays of a patient do not affect those values.

*Areas and Transport* A patient often passes through different locations in a hospital and departments between the various process steps. The scenarios described above can also affect these transport routes and areas within the hospital caused by an attack or failure. For example, house and elevator controls can be affected, leading to considerable additional patient care and transport efforts. In addition to technical attacks, medical reasons, such as quarantine, can also lead to a blockage or failure and impair the process. For these reasons, areas and transport routes are considered in the simulation by defining different areas and the transition duration in regular operation.

**Fig. 1:**
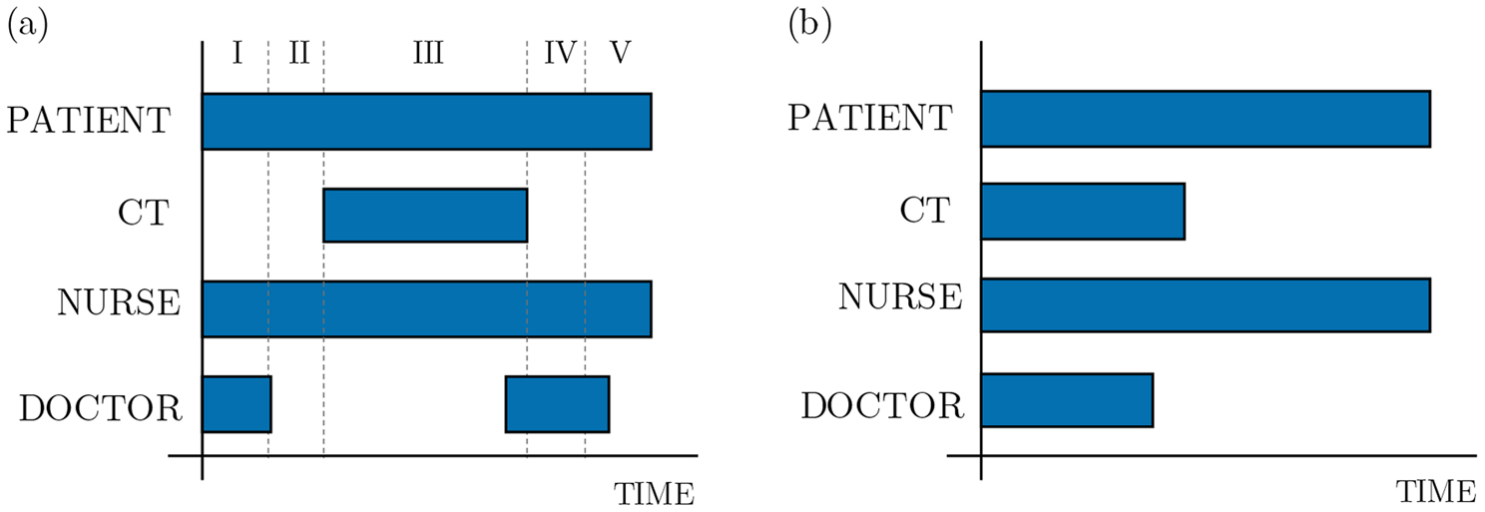
Subfigure (**a**) shows the real-world timings of a Computed Tomography Scan with every resource needed. In (**b**), a simplified time sequence is used

*Resources* The needed and available resources of a process include medical equipment and employees. Each process step defines several resources that are required to perform it. Furthermore, the time a resource is bound to a process step is defined. For example, Fig. 1 shows a simplified time sequence of a Computed Tomography (CT) scan. It is divided into two subfigures, (a) and (b). Both show a time sequence of the physician, the nurse, the CT scanner, and the patient. The first subfigure (a) shows the exact sequence, which allows a very fine granular simulation but increases the configuration effort enormously. The sequence (b) is simpler in terms of configuration but less precise in simulation. Here, the times for the resources and the patient are tied up and run in parallel.

The preferred model can be selected depending on the simulation’s desired level of detail and the available information. Separately from the definition of the required resources, the available resources are set in the respective areas. All processes located in the same area can now access these available resources. Furthermore, a minimum stock level can be set. If this is the case, an area can request devices from other areas, as long as they are portable and above the minimum stock level. The transfer of resources between different areas takes the transition time between the areas into account.

Different resource types categorize technical and medical devices: Devices are divided into portable and stationary devices. Stationary equipment includes, in particular, large-scale equipment such as a CT scanner or Magnetic Resonance Imaging (MRI) scanner. With portable devices, a further distinction is made between devices that become available when the patient moves on to the next process step and devices that remain with the patient until a certain process step has been completed (e.g., a mobile ventilator that stays with the patient from the intubation to the surgery). The portable devices remaining with the patient change the area with the patient and are returned to the original area as soon as the patient no longer requires them. The transition times between the areas are taken into account as well. Further examples of portable devices are infusion pumps and vital monitoring systems.

Employees are also counted as resources in the simulation, as they behave in approximately the same way. The employees are grouped into categories using skill classes. For example, different skills are required to stop bleeding than performing an MRI. Thus, similar to resources, employees of a certain skill level can be requested for a certain time for each process step. Each area has a stock of on-site employees and a minimum stock level that must not be undercut. If there is a shortage, employees can be brought in from other areas; again, the transition period between areas applies. Additionally, there is a special area for alerting physicians who are not on duty. Here, the hospital’s average travel time is used as the transition time to any other area.

Areas can be blocked or quarantined for medical reasons and cannot perform any tasks while in this status. In case of a blocked area without a quarantine, for example, a local blackout, other areas can request resources. If an area gets quarantined, all resources are blocked inside the area and cannot be requested or used by other areas.

*Redundancy* Since the processes under consideration are critical for the outcome of patients, there often are redundant paths. This ensures a successful treatment of patients even if the original path collapses or is overcrowded.

However, such alternative paths are often more inefficient than the main ones, as additional transport and resources are required, and mistakes are more likely to happen [14]. Therefore, the model provides a possibility to mark paths as redundant. In this case, the shortest path in terms of time is selected for each patient depending on the estimated time until the completion of the redundant part.

*Performance Indicators* This model aims to measure process performance under specific conditions focused on the patient’s outcome that may correlate with the delay [38]. The simplest way to specify the performance is the calculation of delays for each patient within the simulation. Before a simulation starts, the expected processing time without any delay is calculated for each patient group, which is marked as “the best pathway”. At the end of the simulation, the difference between the calculated value and the actual duration within the situation is computed. This delay is used as a general indicator of process performance.

A more complex and, therefore, more detailed way to measure the performance is using each patient’s TTD and TTR values. If these values are configured within the simulation, the number of damaged patients or patients requiring resuscitation due to process delays is the process performance indicator.

### Optimizations

Simulation results can be optimized by including real-world data during process configuration. That data can be acquired using standards and default procedures. Furthermore, data sets, including real-world data, can be used. For example, for emergency processes, a German data set called TR-DGU exists. The TR-DGU was founded in 1993. Nearly 700 German and international hospitals participate by providing their data of emergency proceedings. In 2021 more than 35,000 patients were documented in this register, resulting in approximately 313,000 patients in the so-called base-collective over the last ten years (2012–2021) [39]. The collected data is acquired by a questionnaire that has to be filled by the leading doctor after each case within the trauma room. It includes pre-clinic, trauma room, subsequent initial surgery, intensive care unit, and discharge.

The data set offers two information categories that we can combine to optimize the simulation. The first category describes the patient’s injuries using the Abbreviated Injury Scale (AIS). The AIS is a medical score to assess injuries introduced and maintained by the Association for the Advancement of Automotive Medicine (AAAM). The AIS defines nine body regions and an injury scale with six severity levels. The body regions are *Head, Face, Neck, Thorax, Abdomen, Spine, Upper Extremity, Lower Extremity*, and *External or other Trauma*. For each of these body regions, an injury severity score is applied. Possible scores in ascending criticality are *Minor, Moderate, Serious, Severe, Critical*, and *Maximum*. The last means not treatable (yet). All patient records inside the TR-DGU have a rating according to this scale. This enables us to categorize patient populations with the same assigned injury group ${G_i}$, which defines equivalent AIS ratings.

The second information category documents the timestamps of many of the performed actions inside the ETR. It includes the time of arrival, X-Ray scans, CT scans, and initial surgeries until intensive care. Combining these timestamps with the categorized patients, we can generate an average time per action for each injury group ${G_i}$.

The simulation of an emergency process can thus be configured with averaged real-world data for each patient group and enables realistic scenario creation. As the data is standardized and can be acquired for a single hospital or in total, it is even possible to customize the timing data for specific hospitals and compare them or simulate larger scenarios, such as mass casualty events with patients delivered to different departments of traumatology.

## Results

### Implementation

We implement this model to provide an easy-to-use application that can be deployed in hospitals to simulate medical processes. With that in mind, the application is designed so that non-technical users can use it intuitively.

The configuration of simulations should be kept as simple as possible. To ensure this, the configuration is divided into multiple steps that can be reused for different simulations.

In the first step, the user defines global parameters that apply to all simulations. These values form the hospital’s basic configuration, which consists of the hospital’s different areas, including the time needed to switch between them, and the types of resources used in simulations. These types are then linked to different areas as templates for the automatic creation of resources later in the workflow. Resources can be configured to be static or portable so that they may not be available in other areas, such as CT scanners. Thus, the resource templates of an area form its inventory.

The second step consists of the process configuration. Our application supports the wide-used *BPMNv2* [40] standard for process modeling, which allows an easy graphical configuration of processes. An example process is shown in Figure A.1 in Appendix A. After importing *BPMNv2* process data, the user creates a task for every element in the process. This task binds an element to an area and defines which resource types are needed to perform the task and for how long. Alternatively, a resource type can be attached to or detached from a patient. Furthermore, a task can increase a patient’s TTD and TTR, forming a therapy instead of a diagnostic task.

The path a patient takes through a process is defined regarding the patient’s injury group $G$. In addition to that, each group holds information about patient prioritization and ranges for TTD and TTR to facilitate the automated creation of patients for a simulation. Every resource type duration defined for a task can be overridden per injury group to improve the detail level further.

In the third step, the hospital and process configurations are used to build scenarios. A scenario combines a defined process with a certain situation like a sudden change in the availability of key applications or systems. Changes in the number of patients in need of treatment are also considered as a situation. These scenarios act as templates for simulations. First, processes are linked to a scenario. The application automatically links all the related areas and their inventories. It is also possible to add additional areas to the scenario, e.g., an area from which additional personnel can be sourced. In the next step, patients are configured. The user can manually create patients or generate them randomly using the TTD and TTR ranges defined in the injury groups. Finally, the linked objects can be modified to simulate different real-world scenarios. Areas can be deactivated or quarantined. Transition times between areas can be modified, and the efficiency of resources can be increased or decreased. All these modifications are linked to the time they occur in the simulation.

Simulations can be duplicated to create simulation variants fast and easily. This could be helpful in a post-mortem analysis of past events, providing information on how an event could have been handled differently, e.g., by adding specific resources. A simulation provides a status, the functionality to test the configuration for completeness, and the ability to start the simulation. At the start of a simulation, the results are calculated using the model described in Section Model definition.

**Fig. 2:**
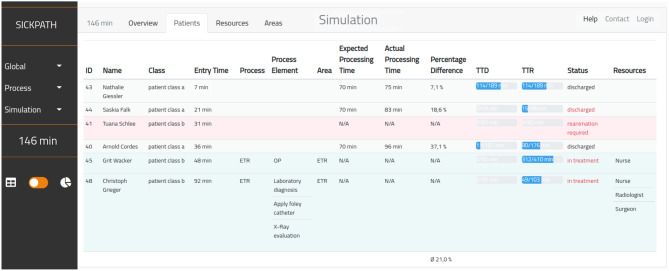
Screenshot of the implemented tool with the results after simulating the ETR process showing the detailed patient information after 146 minutes

The execution of simulations is decoupled from their configuration. This allows users to start a simulation and return to view the results later. After completing a simulation, these results can be retrieved dynamically for every time step of the simulation. Information about patients, resources, areas, and processes can be displayed as summarized graphs or detailed tables. For example, Fig. 2 shows the simulation results based on the ETR process with detailed patient’s information after 146 minutes. Each patient is shown with the actual process, with the current process element, its area, and the currently attached resources. The expected processing time can be compared to the actual processing time if a patient goes through the whole process. Further, the TTD and TTR of each patient are shown in percentage bars and their absolute values in the background. If a patient took damage during the process, the status is labeled in red. This User Interface (UI) design facilitates the evaluation of results and enables the comparability of the simulated scenarios.

All configuration options are determined when a simulation is initiated. This results in the repeatability of simulations as two identically configured simulations will yield identical results.

### Use case demonstration

The process of patients being treated at an ETR is highly standardized and well documented [41]. We use such a process to demonstrate the functionality of our model and simulation tool in three different applications.

#### Preparation

The process, the corresponding hospital configuration, and the patient groups must be defined before the simulation can be performed.

*Process of an ETR* Our ETR process is modeled from a real process at the UKM in Germany. It consists of 26 tasks in five areas: Emergency Trauma Room (ETR), ambulance, Operation Room (OR), Intensive Care Unit (ICU), and Computed Tomography (CT). It covers patient care starting from registration by the rescue service and ending with surgery or a stable situation in the intensive care unit.

In the following, we provide an overview of the process. The full description is listed in Appendix A.

As soon as the patient arrives at the prepared ETR, a trauma surgeon, a general surgeon, an anesthetist, and a radiologist are ready for the patient’s treatment. Depending on the patient’s condition further physicians and nurses are called in. The patient will be treated in three phases, starting with a first survey to stabilize the two most critical body functions, breathing and circulation. In the second survey, intravenous accesses are deployed, and medication and volume therapy are started. Furthermore, x-ray scans of the most important body parts are done. In the last step, a catheter is applied, and the radiologic images are analyzed before the patient is transferred to the CT scanner. The process ends with the transport either to the Intensive Care Unit (ICU) or to the surgery.

*Patient groups* We had access to an anonymized data set from the TR-DGU (see Section Optimizations) of the UKM, including 3044 patients from 2009 to 2018. Using this data set, we generated six patient groups with an equivalent AIS score, which have an average number of 47 data sets per group. This real-world data enables us to configure the duration and the specific path for each patient group using real-life data.

*Simulation parameters* An emergency room setting contains standardized and well-defined resources [42]. These types can be grouped into three categories: Active diagnostic devices, active therapeutic devices, active monitoring devices and consumable supplies (1), premises (2), and human resources (3).

1. Emergency patients within a trauma room have to be diagnosed as accurately and as fast as possible because they often suffer from internal bleeding and fractures that could cause a terminal condition. For this procedure, radiological and sonographic imaging availability are critical key factors. A conventional, portable X-Ray scanner is used to perform the thorax survey to check. This system needs to be portable to enable the staff to perform the imaging and then exclude the system from the limited space to create higher usability.

To perform perfusion scans, the staff needs access to an ultrasonic scanner that supports Doppler sonography. Further, a CT scanner should be available within the trauma section, where the patient can be diagnosed. Controlling the patient’s vital parameters and recognizing any anomaly, every ETR needs to be equipped with monitoring devices capable of measuring ECG, blood pressure, oxygen saturation, and temperature. Additionally, a blood analysis system is required to control the patient’s blood parameters. A medical ventilator and an aspiration device must be available for any breathing problem. For any form of anesthetic procedure, an anesthesia machine is required. Additional essential therapeutic devices are: Infusion pumps are key to stabilizing the patient and applying essential medication like adrenaline. Further, an external defibrillator is required to terminate a potential arrhythmia. Several digital services are required to provide all medical data for the staff. For example, a Picture Archiving and Communication System (PACS) stores all radiological image data and ensures this image data’s availability on every workstation throughout the hospital, whereby a Laboratory information system (LIS) stores all patient laboratory data.

(2) The ETR itself should provide sufficient room for all actions taken. The ER should be near a CT and MRI scanner system, a helipad, and a surgery room for further measures to reduce transport time from one place to another.
(3) The standard ETR team consists of staff from different departments. The base team consists of a trauma leader, a junior surgeon, a senior anesthetist, three members of the nursing staff (two surgical and one anesthetic), and one radiographer. Additionally, this team should be extended by a radiologist and further physicians (ideally 2–3 surgeons). In dependence on the patient’s injuries further senior members of other departments (e.g., neurosurgery or cardiology).

#### Scenario evaluation

To demonstrate the described model’s capabilities, we looked at two different cybersecurity scenarios and one MCI scenario to demonstrate the generic applicability. Each includes a couple of different simulations changing the process and hospital configuration. These simulations show the difference between the standard process duration (baseline) and the simulated scenario.

*Baseline* Each simulation is conducted using twelve patients of the six patient groups derived from the TR-DGU, two for each group. To get a baseline for comparison, a base simulation is created. Within this simulation, one patient from each group is sent through the process one at a time. This ensures that every patient has all resources directly accessible and gives us the treatment duration under optimal conditions. The resulting base duration can be seen in Fig. 5 as *Baseline*. If not defined in the scenario itself, we provide a realistic pattern for the arrival of patients in the hospital based on the measured times of the past.

*Scenario 1: The impact of an acute ransomware attack on the medical imaging system* In the recent past, attacks on the imaging infrastructure of healthcare facilities were discovered [43–45]. The most popular example is the WannaCry ransomware attack that infected and disrupted services of one-third of all trusts of the National Health System in the UK [43]. Once the hospital’s medical imaging system is out of order due to a cyberattack, many processes inside the hospital begin to stall. We apply this global scenario to the specific ETR-process. Without the ability to access medical imaging data of the patient, the staff cannot verify their hypothesis of the patient’s injuries, leading to a decrease in the patient’s chance of survival [46, 47]. As a result, restoring the system’s availability as fast as possible is very important. There are two ways to achieve this after a ransomware attack: Restoring a backup to your systems or paying the demanding ransom to the attackers hoping that they will provide the keys for decrypting the data. Although the first way is preferred, some hospitals paid the ransom as the restoration process would have taken too long or due to faulty or incomplete data backups [44].

The following results show that such a scenario simulation for a fully configured real-life process of a hospital enables decision-making to find critical outage duration to improve their business continuity management. For example, it provides the ability to gather information on tolerable downtimes for business continuity management in addition to holding large-scale emergency exercises. This enables the management to make informed decisions during cybersecurity incidents like ransomware attacks regarding acceptable recovery times.

**Fig. 3:**
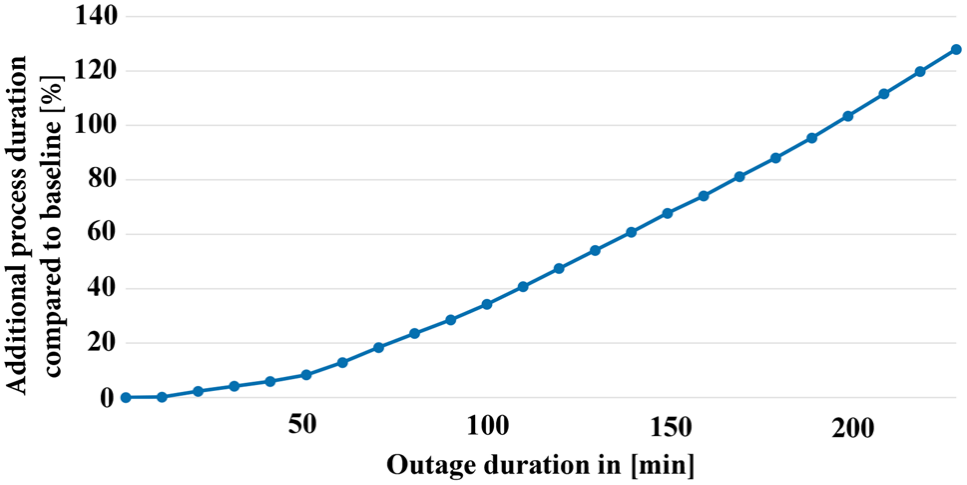
The process duration increases with an increasing CT outage duration. We see a small increase in the process duration from 0–50 min; After this we see a stronger linear growth 0,6 % per minute outage

For planning and decision-making, it may be interesting to find a time frame in which a medical imaging system like a CT has to be restored or have emergency patients driven to other hospitals to minimize the effects of such an attack on the patient. Sometimes, after a ransomware attack, parts of the hospital network are presumable still functional and imaging systems are available in stand-alone mode. For our simulation, we predict the worst case with a total loss of function in these devices. E. g. some imaging system were vulnerable to the infamous CVE-2017-0143 Windows SMB RCE Vulnerability (WannaCry) [48]. Therefore, a series of simulations are conducted with an outage of the CT scanner for a rising duration. The results can be seen in Fig. 3 and show that an outage of up to 30 minutes within our scenario does not affect the average patient flow notably (less than a 10% process duration increase). After 120 minutes of an outage, the process is already 47% less efficient. After 230 minutes, the duration of the process has increased by up to 128%, a value that most probably results in negative effects on the patients’ health.

*Scenario 2: Implementation of security measures* In the past years, the number of cyberattacks has increased continuously [49]. As a result, more and more security measures are implemented by companies [50]. Since medical devices are often designed to work on hardware that is just about sufficient for the task they are built for [51], any software update of such systems might decrease the performance. However, even on powerful devices, security measures often introduce usability problems by restricting features and enforcing security policies [2]. Therefore any security update or security measure can decrease device performance or a prolonged and counterintuitive device handling. Especially in emergency treatment, it is essential that the staff can use those systems as fast and intuitively as possible to treat the patient without any delay that might reduce the hospital care quality and the patient’s well-being [2, 47]. As a result, security measures in emergency treatment must be assessed regarding their cost-benefit factor. For example, measures that do not affect performance, such as network security measures like network separation or implementing SIEM applications, should be preferred.

**Fig. 4:**
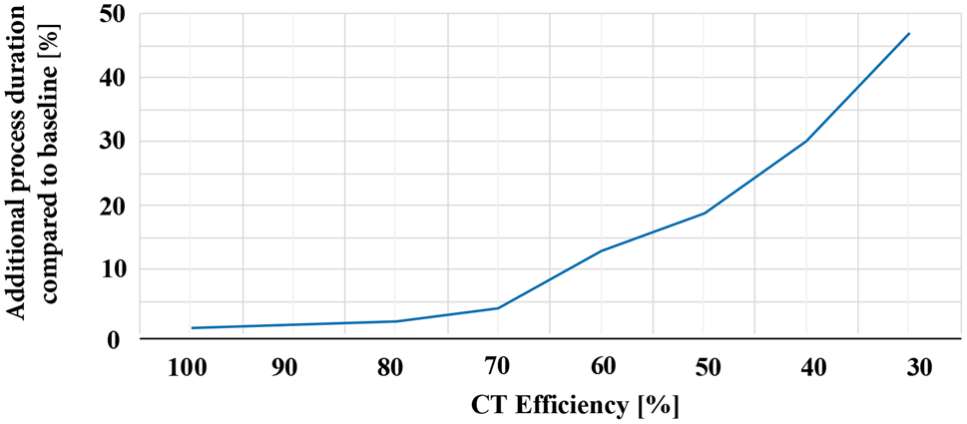
A decreasing CT efficiency results in a process duration increase. We see a small linear growth until 80% followed by a stronger not constant growth until 50%, ending with a strong growth of 1,4 % additional process duration per % decreasing CT efficiency

Within this scenario, the CT scanner described above loses efficiency due to a security measure that prolongs the time required for a CT scan. The process’s performance is measured using four simulations with 100%, 80%, 60%, and 40% efficiency of the CT scanner. As seen in the results visualized in Fig. 4, an efficiency as low as 80% does not affect the overall process efficiency notably. However, efficiencies lower than 80% result in significantly worse process performance compared to the baseline. The ability to simulate the impact of a security measure within a process helps risk management to assess the cost-benefit factor of specific security measures. This enables informed decision-making and may positively affect patient safety.

*Scenario 3: Emergency plans for a Mass Casualty Incident* During a MCI, emergency plans are activated to improve the performance of the ETR and maximize patient safety [52]. In this non-security related scenario, we assume an emergency plan capable of increasing the efficiency of available ETR by adding additional resources such as medical devices and staff.

**Fig. 5:**
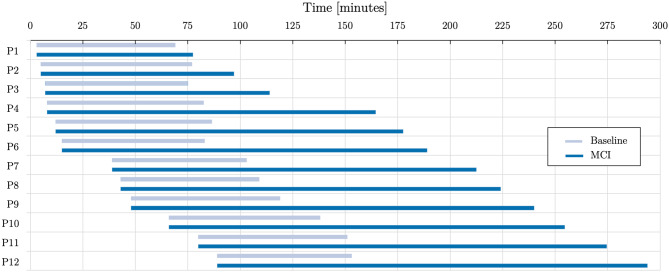
The normal process duration (baseline) is compared to the process duration times of the twelve patients in the MCI scenario

For simulating a MCI, the twelve patients arrive within two batches of six patients each. The first batch of patients arrives between minute three and 15, the second between minute 39 and 89. This scenario leads to an overload situation within the simulated hospital visualized in Fig. 5. This figure shows how every arriving patient increases the stress on the available resources resulting in a maximum delay between a normal ETR-processed patient and one in a MCI of 141 minutes. Comparing the MCI and the baseline data shows an average process time of ${\overline t _{MCI}} = 158.7{\kern 1pt} min$ for the MCI scenario, compared to ${\overline t _{Base}} = 69.3{\kern 1pt} min$ for the baseline.

Based on this overload situation, it is possible to simulate emergency plans and compare their effectiveness. We simulate an emergency plan capable of increasing the effectiveness of all actions within the trauma room by providing more resources and areas for patient treatment. However, the effectiveness of the CT cannot be improved as only one CT is available that is already working at its highest capacity. Finally, we repeated the simulation for the emergency plan but now with a second CT scanner.

**Fig. 6:**
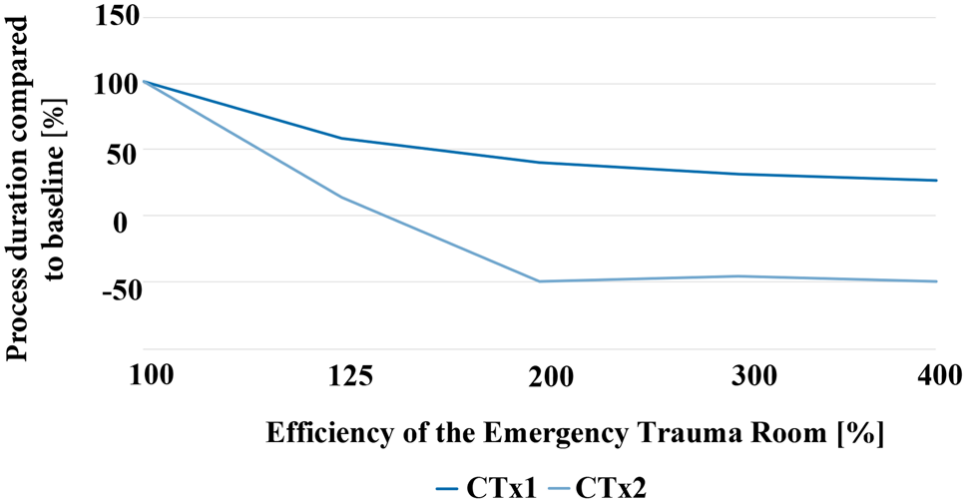
An increase of the ETR efficiency with one and two CT scanners

Our simulation results are visualized in Fig. 6. They show that an increase of the trauma room efficiency of up to 25% significantly improves the overall process performance, up to nearly 50% less additional patient treatment duration than the normal ETR process plan. However, above that value, not much improvement can be seen as there is only one CT scanner in the process. Using a second CT scanner, an overall efficiency increase of up to 200% reduces the patient treatment duration by up to 150%, which means that the patients are treated even faster than in a non-MCI situation. The graph shows that this 200% is the limit of the improved efficiency due to the maximum workload of both available CT scanners.

This sample simulation shows the model’s ability to compare different emergency plans to identify components that highly influence the overall process performance in overload situations. Additionally, it is possible to compare ETR of different hospitals with each other for better capacity planning or optimizing patient routing during MCI events.

## Discussion

From the authors’ point of view, our simulation can never replace practical and past experience. It is, therefore, only possible for us to calculate a possible outcome on the basis of past events and the measured values obtained from them. The real applicability of our modeling remains to be determined since, in our paper, we have only examined one particularly standardized and widespread process, which is also historically very precisely surveyed and trained. Therefore, a limitation of our publication is the requirement that clinical processes are both noted and run in the same way. The latter, in particular, is often not the case in reality. One can often find process notations of varying quality and depth. During our mining processes, we were able to access extensive data sets and conduct expert interviews. We assume this would not be possible for lesser standardized processes. These conditions are only available to a limited extent for other processes. Another limiting factor is the problem of subsequent documentation. In our interviews, it became clear that even with our measured values, the members of the process often only come together to document the process after it has been completed. There is a risk that times and processes cannot be recorded accurately due to the time difference. Reviewing our results and discussing them with the clinical staff, we encountered some discrepancies due to individual dependencies based on local circumstances like micro changes in the clinical pathway in order to adapt to the individual patient condition. As we used a Python-based scripted approach in combination with elastic search, there are limitations in the form of performance. We tried to overcome this issue with parallelism. For a more capable version of our tool, this could be improved. Furthermore, a cyber attack is only one possible reason for a disruption of healthcare processes in a hospital. When reviewing our data, we also consulted the hazard catalogs of the usual standards and the B3S [1]. When planning incident response, our publication and the underlying tool would also provide added value here.

## Conclusions

Within this work, we present a hybrid model of DES and ABS capable of performing patient-focused simulations of processes inside a hospital environment to support risk management. The model can be used for simulations with different levels of detail. While a low level of detail gives basic results, adding more information into the model enables simulations close to reality. It further includes the ability to add historical data to adapt the simulation to a specific hospital and for retrospective analysis of incidents. To explore the model’s accuracy we were able to pilot the analysis model. We performed different scenario simulations for a sample hospital with a focus on an ETR: The short-term consequences of a ransomware cyberattack on the medical imaging system, negative impacts due to the implementation of cybersecurity measures, and emergency plans for a mass casualty incident.

The results of the first scenario enable decision-makers to estimate the maximum tolerable downtime of an imaging system during a cyberattack and the recovery phase. Such information allows estimations about countermeasures and recovery strategies, which are essential for expensive and serious decisions, e.g., the deregistration from trauma care due to the simulated possible outcomes of patients. The second scenario provides information about possible negative effects of cybersecurity measures that lead to prolonged processes. The simulation of the last scenario provides insight into the impact of emergency plans during an MCI and shows the generic applicability of our model.

The model introduced in this work serves as a basis for informed decision-making within hospitals. It is ready to get further improvements by assessing it in real-world environments, including realistic TTD and TTR data for the generated patients as well as the data of the TR-DGU. Different ETR can be compared to each other. Simulating of real-world events can be performed as a retrospective analysis to get further adjustments to increase the reality level even more. With this further development, the model may improve emergency planning for exceptional situations and reduce patient safety risks in the future.

## Electronic supplementary material

Below is the link to the electronic supplementary material.

Supplementary Material 1

## Data Availability

Raw anonymous data are not publicly available under the TR-DGU and UKM approval terms. Access to the TR-DGU data for research projects is subject to the requirements of the TR-DGU. The developed simulation tool is not openly available for license reasons and is available from the corresponding author upon request.

## Abbreviations

AAAM: Association for the Advancement of Automotive Medicine
ABS: Agend-Based Simulation
AIS: Abbreviated Injury Scale
BCM: Business Continuity Management
CT: Computed Tomography
DES: Discrete Event Simulation
ETR: Emergency Trauma Room
FDA: U.S. Food and Drug Administration
ICU: Intensive Care Unit
LIS: Laboratory information system
MCI: Mass Casualty Incident
MRI: Magnetic Resonance Imaging
PACS: Picture Archiving and Communication System
TR-DGU: TraumaRegister DGU
TTD: Time till Damage
TTR: Time to Reanimate
UI: User Interface
UKM: Muenster University Hospital
