# Supplementary material for: Simulating the overload of medical processes due to system failures during a cyberattack
Source: BMC Med Inform Decis Mak. 2025 Apr 23;25:174. doi: 10.1186/s12911-025-02988-8 (PMC12016121; doi:10.1186/s12911-025-02988-8)
Supplement: Supplementary file 1 — Supplementary Material 1 [file 12911_2025_2988_MOESM1_ESM.pdf]

Appendix A: Our modeled process of an Emergency Trauma Room (ETR)

Our Emergency Trauma Room (ETR) process (cf. Figure A.1), is modeled from a real process at the Münster University Hospital (UKM), Germany and consists of 26 tasks in five areas. The following description includes the required resources and the default timings for each action.

The process starts with a call from the emergency physician on-scene, answered by a trauma surgeon. This standardized call takes about 60 seconds and informs the trauma surgeon about the basic questions *What*, *Who*, and *How*, the estimated time of arrival (ETA), if the transport is ground- or airborne, if the patient is awake and responsive, and if they are stable or unstable. After that, the trauma surgeon calls the responsible coordinator, who informs the necessary staff. This call takes approximately another 60 seconds. The following five minutes are used to prepare the ETR and ensure all necessary resources are available.

Five minutes before the ETA of the patient, the staff meets inside the ETR for a short briefing and preparation. As soon as the patient arrives, a short handover is performed, and the patient is undressed and repositioned to the treatment table of the ETR. After that, the time measurement inside the room starts, and three steps of parallel tasks are performed following the ABCDE (**A**irway, **B**reathing, **C**irculation, **D**isability, and **E**xposure) approach.

The first step of four parallel tasks aims to assess Airway, Breathing, and Circulation problems and solve them to keep the patient stable. The trauma surgeon and their assistant are responsible for checking the cervical collar or install one (30 seconds). Furthermore, they perform circulation check-up on the Thorax, Abdomen, and Thighs (60 seconds). In case of circulation problems, several surgical emergency procedures could be initiated with an overall duration of up to thirteen minutes. These procedures include the surgical opening of the thorax (Thoracotomy) or the abdomen (Laparotomy) and the installation of a bleeding suppression (Tourniquet). The anesthetist and her assistant require three minutes to perform an airway check, auscultation of both lungs, and basic monitoring installation, including blood pressure, pulse, saturation, and Electrocardiogram (ECG). If there is a problem regarding the airway, an emergency procedure taking 5 minutes is initiated, including either the patient's intubation or an emergency opening of the trachea (cricothyrotomy). If the patient suffers from insufficient breathing, a ventilation system is installed by the anesthetist, and an emergency procedure can be initiated to set a chest drain taking another 10 minutes. Simultaneously, the radiologist or the general surgeon performs a rapid ultrasound examination of the patient.

After the first parallel tasks were performed, the so-called Secondary Survey is started, again in parallel. The trauma surgeon performs the rest of the body check (four minutes), and the trauma nurse checks the tetanus vaccination status. The anesthetists initiate two intravenous accesses and start with medication and volume therapy. Furthermore, they take care of properly adjusted ventilation, taking 5 to 10 minutes. Additionally, it takes five minutes for the radiographer to x-ray the thorax and the pelvis. Simultaneously, the patient's level of consciousness is measured using the Glasgow Coma Scale (GCS) by giving points in the three categories Eye, Verbal, and Motor response.

In the third and last step inside the ETR, a Blood Gas Analysis (BGA) is performed, a urinary catheter is applied, and the x-ray images from the previous step, as well as the results of the BGA are analyzed. After the third step is completed, the patient is transported to the Computed Tomography (CT) scanner. They are escorted by the trauma surgeon with the assisting nurse, the anesthetist with the assisting nurse, the radiologist, and the radiographer. The transport takes about three minutes. Arriving at the CT scanner, the patient is relocated to the CT scanner table, and several CT scans are performed, including a special trauma protocol. These scans take approximately 23 minutes and include a computed tomography of the head and cervical spine and a Whole-body computed tomography (WBCT). Depending on the information available, the staff decides if further X-ray scans are necessary (five to ten minutes). Afterward, the patient is transported to the surgery room or the Intensive Care Unit (ICU).

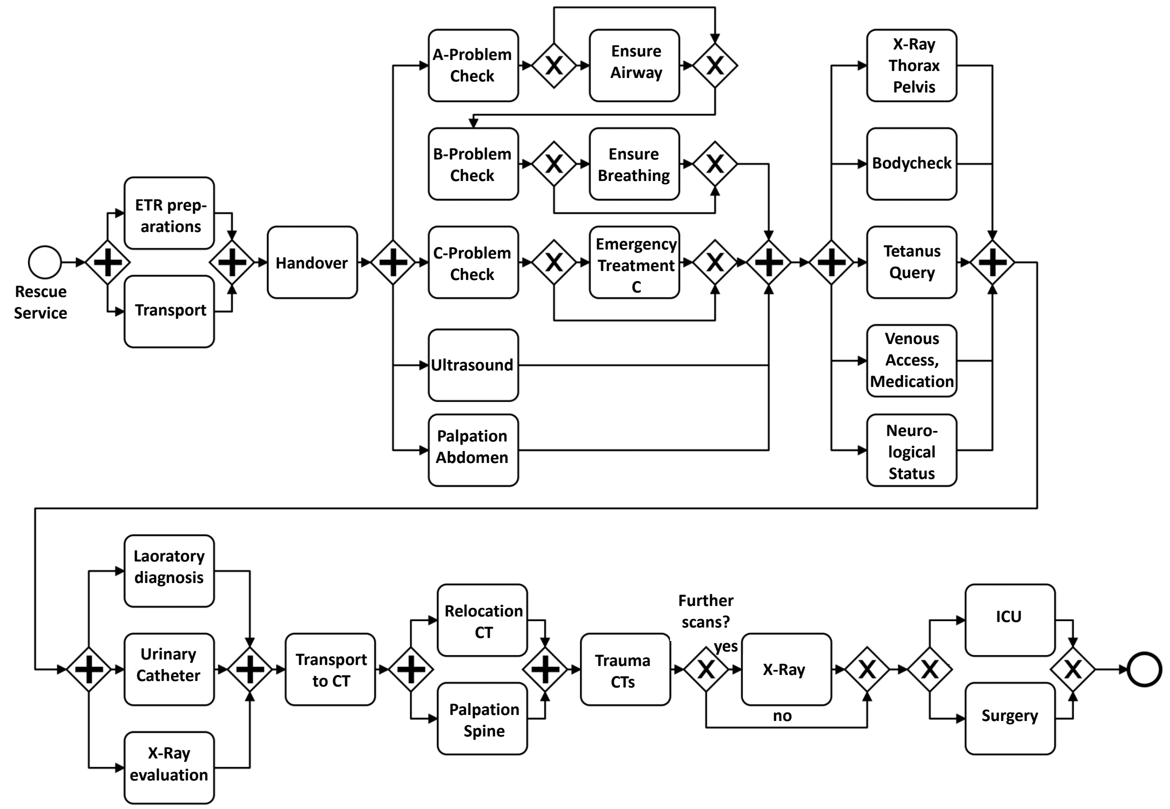

Figure A.1: The modeled ETR process from the arrival of the rescue team till the handover to the ICU or surgery.
